# Performance of PSMA-targeted radiotheranostics in an experimental model of renal cell carcinoma

**DOI:** 10.3389/fonc.2024.1432286

**Published:** 2024-09-10

**Authors:** Rajan Singh, Anand K. Thotakura, Suresh Alati, Alla Lisok, Zirui Jiang, Vanessa F. Merino, Il Minn, Santosh Yadav, Mark C. Markowski, Yasser Ged, Christian P. Pavlovich, Nirmish Singla, Lilja B. Solnes, Michael A. Gorin, Martin G. Pomper, Steven P. Rowe, Sangeeta Ray Banerjee

**Affiliations:** ^1^ Russell H. Morgan Department of Radiology and Radiological Science, Johns Hopkins University, Baltimore, MD, United States; ^2^ Department of Oncology, Sidney Kimmel Comprehensive Cancer Center, Baltimore, MD, United States; ^3^ Department of Urology, Brady Urological Institute, Johns Hopkins University, Baltimore, MD, United States; ^4^ The Milton and Carroll Petrie Department of Urology, Icahn School of Medicine at Mount Sinai, New York, NY, United States; ^5^ Department of Radiology, UT Southwestern Medical Center, Dallas, TX, United States; ^6^ Department of Radiology, University of North Carolina, Chapel Hill, NC, United States

**Keywords:** prostate-specific membrane antigen, positron-emission tomography, gallium-68, lutetium-177, actinium-225, targeted radiopharmaceutical therapy, alpha-particle emitting radionuclide, β-particle emitting radionuclide

## Abstract

**Introduction:**

Renal cell carcinoma (RCC) represents cancer originating from the renal epithelium and accounts for > 90% of cancers in the kidney. Prostate-specific membrane antigen (PSMA) is overexpressed in tumor-associated neovascular endothelial cells of many solid tumors, including metastatic RCC. Although studied in several small clinical studies, PSMA-based imaging and therapy have not been pursued rigorously in preclinical RCC. This study aimed to evaluate the preclinical performance of PSMA-based radiotheranostic agents in a relevant murine model.

**Methods:**

A PSMA-overexpressing murine cell line, PSMA+ RENCA, was developed by lentiviral transduction. PSMA-based theranostic agents, ^68^Ga-L1/^177^Lu-L1/^225^Ac-L1, were synthesized in high radiochemical yield and purity following our reported methods. Immunocompetent BALB/c mice were used for flank and orthotopic tumor inoculation. ^68^Ga-L1 was evaluated in small animal PET/CT imaging in flank and PET/MR imaging in orthotopic models. Cell viability studies were conducted for ^177^Lu-L1 and ^225^Ac-L1. Proof-of-concept treatment studies were performed using ^225^Ac-L1 (0, 37 kBq, 2 kBq × 37 kBq, 1 week apart) using PSMA+ RENCA in the flank model.

**Results:**

Cellular uptake of ^68^Ga-L1, ^177^Lu-L1, and ^225^Ac-L1 confirmed the specificity of the agents to PSMA+ RENCA cells rather than to RENCA (wt) cells, which are low in PSMA expression. The uptake in PSMA+ RENCA cells at 1 h for ^68^Ga-L1 (49.0% incubated dose [ID] ± 3.6%ID/million cells), ^177^Lu-L1 (22.1%ID ± 0.5%ID)/million cells), and ^225^Ac-L1 (4.1% ± 0.2% ID)/million cells), respectively, were higher than the RENCA (wt) cells (~ 1%ID–2%ID/million cells). PET/CT images displayed > 7-fold higher accumulation of ^68^Ga-L1 in PSMA+ RENCA compared to RENCA (wt) in flank implantation at 1 h. A twofold higher accumulation of ^68^Ga-L1 was observed in orthotopic tumors than in normal kidneys during 1–3 h postinjection. High lung uptake was observed with ^68^Ga-L1 PET/MR imaging 3 weeks after orthotopic implantation of PSMA+ RENCA due to spontaneous lung metastases. The imaging data were further confirmed by immunohistochemical characterization. ^225^Ac-L1 (0-37 kBq) displayed a dose-dependent reduction of cell proliferation in the PSMA+ RENCA cells after 48 h incubation; ~ 40% reduction in the cells with treated 37 kBq compared to vehicle (*p* < 0.001); however, no effect was observed with ^177^Lu-L1 (0–3700 kBq) up to 144 h postinoculation, suggesting lower efficacy of β-particle-emitting radiations in cellular studies compared to α-particle-emitting ^225^Ac-L1. Animals treated with ^225^Ac-L1 at 1 week posttumor inoculation in flank models displayed significant tumor growth delay (*p* < 0.03) and longer median survival of 21 days and 24 days for the treatment groups 37 kBq and 2 kBq × 37 kBq, respectively, compared to the vehicle group (12 days).

**Conclusion:**

The results suggest that a theranostic strategy targeting PSMA, employing PET and α-emitting radiopharmaceuticals, enabled tumor growth control and enhanced survival in a relevant immunocompetent murine model of RCC. These studies provide the rationale for clinical studies of PSMA-targeted theranostic agents in patients with RCC.

## 1 Introduction

Renal cell carcinoma (RCC) comprises ~ 3.8% of all malignancies, with an estimated 81,610 new cases annually, with 14,390 patients who will die from the disease in 2024 in the USA (1). Nearly one-third of patients have metastatic disease at initial presentation, and 30%–50% of the patients treated for localized RCC will subsequently develop metastases. There has been considerable progress in the treatment of RCC since 2005 with a variety of agents, including those targeting the vascular endothelial growth factor (VEGF)/receptor (VEGFR) axis, mammalian target of rapamycin signaling (mTOR) pathways, immune checkpoint inhibitors (ICIs) and most recently, through combination of antiangiogenic agents and ICIs or employing a dual ICI strategy (2, 3). Nevertheless, the overall low response rates of those newer therapies necessitate a continued search for more definitive treatment, which might be achieved by a targeted radiotheranostic approach.

PSMA represents a validated biomarker for targeting the neovasculature of several epithelial malignancies, including metastatic clear cell RCC (mRCC), the most common and lethal subtype (4–10). Multiple studies have demonstrated the utility of PSMA-targeted PET imaging (PSMA PET) in mRCC, particularly for the clear cell subtype, using the low-molecular-weight agents ^18^F-DCFPyL and ^68^Ga-PSMA-11 (10–20). The sensitivity of PSMA PET for sites of mRCC is substantially higher than conventional imaging with contrast-enhanced CT or MRI (94.7% vs. 78.9% in a five-patient series), suggesting that even sites of minimal disease have significant PSMA-mediated radiotracer uptake (12). The high sensitivity and specificity of PSMA PET agents in this context suggest that PSMA-based radiopharmaceutical therapy may enable the treatment of RCC with relatively low off-target effects, as demonstrated by this approach used for prostate cancer (21). One study indicated that PSMA expression portends an unfavorable clinical outcome in patients with clear-cell RCC (*n* = 257) (22). Those cumulative data support the development of PSMA-targeted radiopharmaceuticals for RCC imaging and therapy (radiotheranostics). Indeed, two PSMA-based radiotheranostic agents entered clinical trials in mRCC (NCT05170555 and NCT06059014), studying the safety and efficacy of β-particle-emitting ^177^Lu-labeled low-molecular-weight agents (23, 24). Furthermore, the α-particle-emitting bone-targeting radiotherapeutic agent, ^223^RaCl_2_, in combination with tyrosine kinase inhibitors (TKIs), was studied in mRCC patients with bone metastases and demonstrated safety and efficacy (25). While the efficacy of ^223^RaCl_2_ is limited to bone lesions, PSMA-based radiotherapeutics can treat lesions anywhere in the body, including bone metastases. We and others have shown that patients with mRCC, resistant to TKIs and immunotherapy with ICIs, displayed high neovascular PSMA expression in metastatic lesions in PET imaging (13, 26). Despite those small case series, a more rigorous preclinical assessment of PSMA radiotheranostics in RCC is lacking.

To determine the feasibility of a radiotheranostic strategy, we selected our previously developed β-particle-emitting low-molecular-weight compound, ^177^Lu-L1, which demonstrated reduced off-target effects in a xenograft model of prostate cancer (27). We leveraged that PSMA-targeting scaffold to develop α-particle-emitting analogs, ^213^Bi-L1, ^225^Ac-L1, ^212^Pb-L1, and ^221^At-labeled analogs of L1 (27–30). An analog of the radiotheranostic platform of ^68^Ga-L1/^177^Lu-L1/^225^Ac-L1 entered a clinical trial recently (NCT05983198). Considering that the PSMA expression of tumor-associated neovasculature is low in most preclinical RCC models, we developed a human PSMA-transduced murine RCC cell, PSMA+ RENCA, as a model for a proof-of-concept preclinical therapy study. We conclude that a PSMA-based radiotheranostic platform using ^68^Ga-L1/^177^Lu-L1/^225^Ac-L1 is a feasible strategy for treating patients with PSMA+ mRCC.

## 2 Materials and methods

### 2.1 Cell lines and animal models

Murine RCC RENCA wild type (wt) (catalog No. 2947) and LNCaP clone FGC (catalog No. 1740) cell lines were obtained from ATCC (Gaithersburg, MD, USA) and grown in RPMI medium supplemented with 10% FCS (Thermo Fisher Scientific, Waltham, MA, USA), 0.1 mmol/L nonessential amino acids (Thermo Fisher), 1 mmol/L sodium pyruvate (Thermo Fisher), and 2 mmol/L l-glutamine (Thermo Fisher). Human prostate cancer PC3 cell lines, PSMA+ PC3 PIP, PSMA− PC3 flu, and LNCaP cells were used as control cell lines and grown following our reported protocol (31). RENCA cells were stably transduced with human PSMA using lentiviral transduction. Five- to six-week-old male/female immunocompetent BALB/c mice from Jackson Laboratory were used for orthotopic (0.2–0.5 × 10^6^ cells) and subcutaneous implantation (2 × 10^6^ cells) for tumor model generation (32).

### 2.2 Radiolabeling

The PSMA-targeting ligand L1 was synthesized following a reported method (27). A known small-molecule Glu-Lys urea-based PSMA inhibitor, ZJ43, was prepared in-house, as reported (33), and used for receptor-blocking studies. Radiolabeling was done following our report (27, 34) and is included in the **Supplementary Material**. High Performance Liquid Chromatography (HPLC) purification of the radiolabeled compounds was performed on an Agilent HPLC instrument (Agilent Technologies, Santa Clara, CA, USA) coupled to a radiodetector (Bioscan - Flow-Count) using a Phenomenex Luna C_18_ HPLC column (00G-4252-E0; 250 mm × 4.60 mm, 5 μL, 100 Å). The purified radiolabeled product (^68^Ga-L1/^177^Lu-L1/^225^Ac-L1) was diluted with saline to the desired radioactivity concentration for imaging and biodistribution studies.

### 2.3 Flow cytometry

PSMA+ RENCA and RENCA (wt) cells (1 × 10^6^ cells) were harvested and stained with PE anti-PSMA antibody (No. 342504, BioLegend, San Diego, CA, USA) following the manufacturer’s protocol. The cells were incubated at 4°C for 1 h in the dark and washed with cold phosphate-buffered saline (PBS). The fluorescence intensities of both unstained and stained cells were analyzed using flow cytometry (Attune NXT), and quantitative data analysis was performed with FlowJo software. PSMA+ PC3 PIP and LNCaP cell lines were used as the positive control, and PSMA− PC3 flu cell was used as the negative control (31). The data were analyzed using FlowJo software.

### 2.4 Receptor density measurements

A Phycoerythrin Fluorescence Quantitation Kit (No. 340495, BD Biosciences, Franklin Lakes, NJ, USA) containing four levels of phycoerythrin/bead was used. Beads were reconstituted with 0.5 mL of PBS containing sodium azide and 0.5% bovine serum albumin before use. Cells were stained for 30 min at 4°C with a phycoerythrin-labeled anti-PSMA antibody and run along with the beads to estimate receptor density using flow cytometry. A calibration curve of geometric mean versus phycoerythrin/bead from different bead populations was generated following the manufacturer’s protocol. This calibration curve was used to derive receptors/cells for each cell type from their respective geometric means. Isotype controls were used to eliminate any nonspecific staining.

### 2.5 Quantitative real-time PCR analysis

The assay was done following a reported method (31). For mRNA isolation, PSMA+ RENCA and RENCA (wt) cells were grown in six-well cell culture plates. cDNA was then synthesized from 1 μg of total RNA per experimental replicate using an Applied Biosystems cDNA synthesis kit following the manufacturer’s protocol (Thermo Fisher). mRNA amplification was carried out on the Applied Biosystems 7500 Fast Detection System (Thermo Fisher) with SYBR green qPCR master mix (Bio-Rad, Hercules, CA, USA), as per the manufacturer’s instructions. All reactions were conducted in triplicate, and negative controls were included in each experiment. GAPDH was the internal control, and data sets were normalized to GAPDH levels. The fold change in gene expression (FOLH1 TaqMan probe; Life Technologies, MD, USA) was determined using the Δ2CT method, and results were reported in arbitrary units or as fold changes. The sequences of the primers will be provided upon request.

### 2.6 Immunoblotting

The immunoblot study (Western blot [WB]) was done following a protocol reported by our lab (35). PSMA+ RENCA and RENCA (wt) were homogenized in RIPA buffer and protein quantified using the Pierce BCA Kit (Thermo Fisher). Membranes were incubated at room temperature with PSMA (Cell Signaling Technology, Danvers, MA, USA, No. D718E) and GAPDH (Cell Signaling Technology, No. D16H1) antibodies. The membranes were incubated with HRP-coupled antirabbit IgG secondary antibodies, and the blots were developed using an ECL reagent. Digital quantification of chemiluminescence was performed using Image J software (NIH).

### 2.7 Cell uptake studies

Cell uptake studies were conducted following our established protocol (36). Briefly, PSMA+ RENCA and RENCA (wt) cells (~ 1 × 10^6^ cells per tube) were detached using a nonenzymatic buffer and were incubated with 100 µL of ^68^Ga-L1 or ^177^Lu-L1 or ^225^Ac-L1 (37 kBq/mL) for indicated time points at 37°C in the growth medium (100 µL). After incubation, the medium was removed at the indicated time points, and the cells were washed with ice-cold PBS. Radioactivity in the collected pooled washes and the cell pellets were counted using an automated γ-counter and expressed as percentage incubated dose (%ID)/million cells. To evaluate PSMA specificity, cells were pre-incubated with ZJ43 (10 µM final concentration) for 30 min, washed with binding buffer, and then incubated with the radiotracer (37 kBq/mL) for the indicated time points. The cell uptake studies were then performed as described above. The data are presented as %ID per million cells.

### 2.8 PET/CT and PET/MR imaging

Whole-body PET/CT imaging studies were acquired on a SuperArgus PET/CT preclinical imaging system (SEDECAL SA2R PET/CT, Madrid, Spain). The tumor-bearing mice were injected with 7.4 MBq ± 0.37 MBq of ^68^Ga-L1 intravenously, anesthetized under 3% isoflurane, and maintained under 1.5% isoflurane (v/v) for imaging studies. Images (two-bed position, 10 min per position) were acquired at indicated time points after ^68^Ga-L1 injection. Imaging data were corrected for decay and dead time and were reconstructed using the three-dimensional ordered-subsets expectation maximization algorithm.

A simultaneous PET/MR scanner with a three-ring Bruker Si198 PET insert (7T preclinical PET/MRI, Bruker BioSpec, 70/30 USR, Ettlingen, Germany) was used for imaging orthotopic tumor models. The mice were injected with 7.4 MBq of ^68^Ga-L1 intravenously, anesthetized, and maintained under 2.0%–1.5% isoflurane (v/v) for the imaging study. Simultaneous whole-body MR imaging and 10-min static PET imaging were performed using a 72-mm PET-observed transfer/receive MR coil centered inside the PET detector. The MR imaging protocol includes a whole-body coronal TurboRARE T2-weighted scan with an echo time/repetition time (TE/TR) of 36 ms/3,000 ms, a field of view (FOV) of 100 mm × 60 mm, 24 slices, a 1-mm slice thickness, a matrix size of 400 × 280, four averages, and a rare factor of 8. We also collected the higher resolution axial TurboRARE T2-weighted scan covering the tumor area with an TE/TR of 36 ms/3,200 ms, a FOV of 28 mm × 32 mm, 20 slices, a 0.7-mm slice thickness, a matrix slice of 120 × 120, six averages, and a rare factor of 8. The data were reconstructed using maximum likelihood expectation maximization (MLEM) with a 0.5-mm preset and 18 iterations. After quality assessment, reconstructed MR imaging data were spatially coregistered with PET data using PV 360, and analysis was performed using AMIDE (https://amide.sourceforge.net/). The final images were displayed using the AMIRA software (Thermo Fisher Scientific).

### 2.9 Biodistribution

The biodistribution study was conducted following our previously reported method (27, 28). Male and female BALB/c mice bearing PSMA+ RENCA flank tumors (right flank) and RENCA (wt) (left flank) (*n* = 4) were administered with ^225^Ac-L1 (37 kBq) in 150 µL of saline via tail-vein injection. Biodistribution studies were conducted at 3–4 weeks after tumor inoculation. A second biodistribution study was conducted using the PSMA+ RENCA orthotopic model (*n* = 4). The mice were euthanized at the indicated time after the injection of ^225^Ac-L1, and blood, tumor, and selected organs (heart, lungs, liver, stomach, pancreas, spleen, fat, kidney, small intestine, salivary gland, lacrimal gland, urinary bladder, bone, and muscle) were harvested, weighed, and assayed for radioactivity using an automated γ-counter 24 h after the sacrifice time to ensure complete secular equilibrium. The percentage of injected dose per gram of tissue (% ID/g) was calculated by comparison with samples of a standard dilution of the initial dose.

### 2.10 Radiopharmaceutical therapy

#### 2.10.1 Cell viability assay

Cell viability was measured using the CellTiter-Glo (CTG) luminescent cell viability assay (Promega, Madison, WI, USA) following the manufacturer’s instructions. Briefly, cells were seeded in a 96-well plate (10,000 cells/well) in 100 µL media. After 24 h incubation, the supernatant was removed, and the cells were treated with either ^177^Lu-L1 (0–3,700 kBq/mL and 100 µL/well, 96 h) or ^225^Ac-L1 at different concentrations (0–37 kBq/mL and 100 µL/well, 48 h). After 48 h of incubation at 37°C, the medium was replaced with a freshly prepared solution of CTG (100 µL/well) and incubated in the dark for 3 h. Subsequently, 100 µL of the supernatant from each well was transferred to a 96-well luminometer-compatible plate, and luminescence was recorded using the POLARstar Omega Microplate reader (BMG Labtech, Taunton, MA, USA). The response readout was normalized to the control (untreated cells).

#### 2.10.2 *In vivo* therapeutic efficacy

Mice were injected subcutaneously with PSMA+ RENCA cells (1 × 10^6^ cells/150 µL HBSS) on the upper right flanks. Mice were injected intravenously with either (a) a single dose (1 week after tumor inoculation, group 1) or (b) two consecutive doses (1 week apart) of 37 kBq × 2, group 2). The control group (group 3) received 150 µL of saline (*n* = 10). The changes in body mass and tumor volumes of animals were recorded two to three times per week. Mouse body weight and tumor volume were monitored every 3 days. The formula used to calculate tumor volume was *V* = width^2^ × length/2. Endpoint criteria, as defined by the JHU animal care and use committee (ACUC), were weight loss ≥ 15%, tumor volume > 1,000 mm^3^, active ulceration of the tumor, or abnormal behavior indicating pain or distress. The survival of mice was assessed with Kaplan–Meier curves to determine the median survival of mice (*n* = 7) in each group. The definitions of endpoint criteria were also used for Kaplan–Meier analysis. On day 8, after ^225^Ac-L1 injection, three mice per group were removed, and tumor sections were prepared for hematoxylin and eosin (H&E) and immunohistochemical (IHC) characterization.

### 2.11 Immunohistochemistry

IHC staining was done as we previously reported (27, 28). Tumor sections were incubated with either Primary antibody anti‐PSMA (1:100 dilution; No. D718E, Cell Signaling, Danvers, MA, USA), primary antibody anti‐CD31 (1:100 dilution; No. PECAM-1, Cell Signaling), and primary antibody anti‐gH2AX (1:100 dilution; No. D17A3, Cell Signaling). Primary antibodies were detected using an anti-rabbit HQ detection system (Roche Diagnostics, No. 7017936001 and No. 7017812001), amplified by Discovery AMP Multimer (Roche Diagnostics, DC, USA, No. 6442544001), followed by the Chromomap DAB IHC Detection Kit (Roche Diagnostics, No. 5266645001).

### 2.12 Statistics

Statistical analyses were performed on Prism software (GraphPad, version 9.0, GraphPad Software, Boston, MA, USA). The Student’s unpaired *t*-test was conducted to determine statistical significance. All tests were two-sided unpaired, and *p*-values of less than 0.05 were considered statistically significant. The cell uptake, tissue biodistribution, and imaging data were presented as mean ± SEM.

## 3 Results

### 3.1 *In vitro* characterization reveals surface PSMA expression in PSMA+ RENCA cells

**Figure 1:**
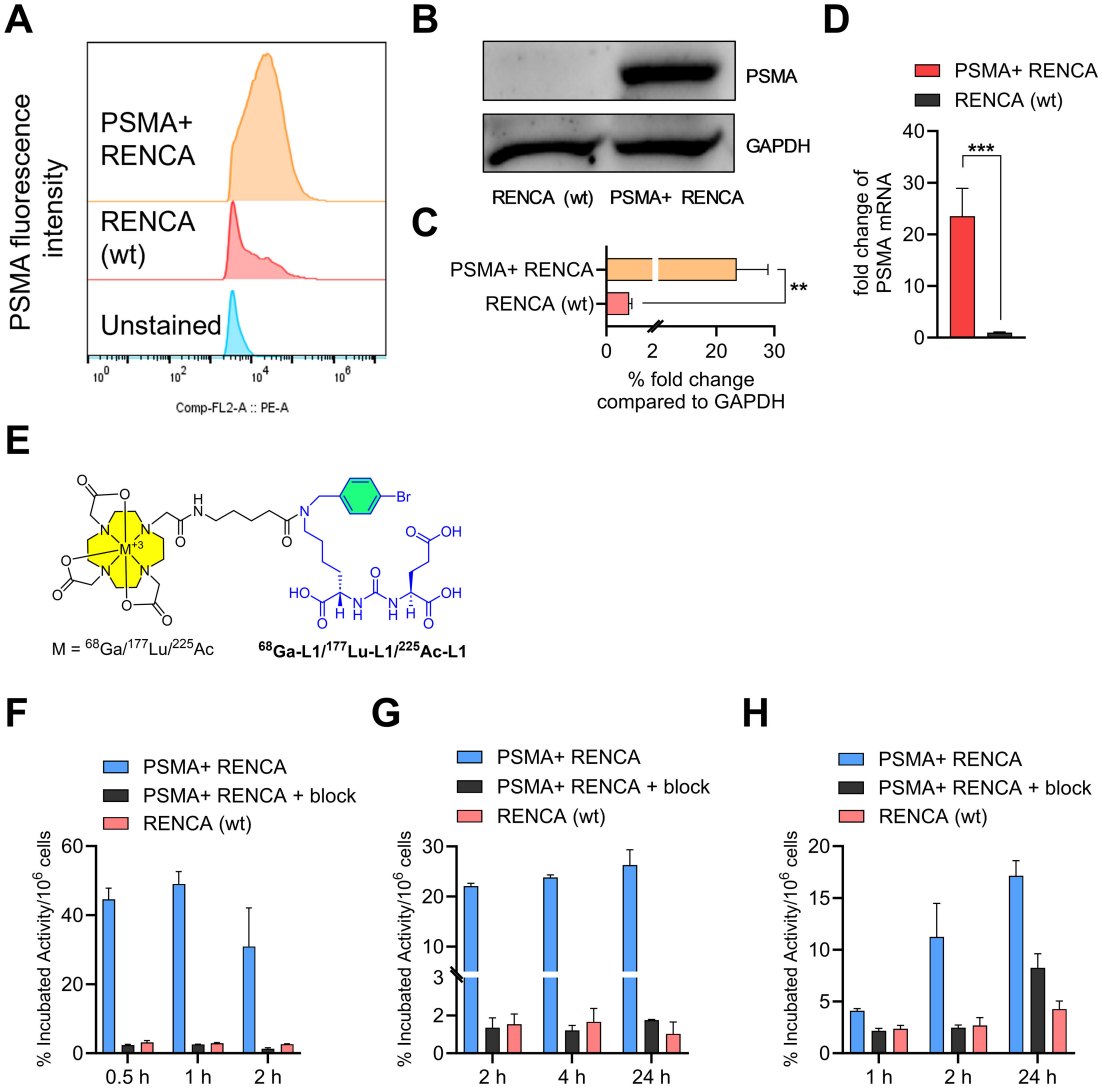
Characterization of PSMA+ RENCA and RENCA (wt) cell lines. **(A)** Flow cytometry of PSMA surface expression. Quantitation of mean fluorescence intensity shows that PSMA+ RENCA cells have higher levels of PSMA surface expression than RENCA (wt) cells. **(B)** PSMA total protein levels in PSMA+ RENCA and RENCA (wt) cells by Western blot. **(C)** Relative fold change in PSMA total protein levels compared to GAPDH. **(D)** PSMA mRNA levels in PSMA+ RENCA and RENCA (wt) cells by RT-qPCR. **(E)** Chemical structure of PSMA-based radiopharmaceutical agents used in this study. **(F–H)** Cell uptake (average ± SD, *n* = 3) of **(F)** ^68^Ga-L1, **(G)** ^177^Lu-L1, and **(H)** ^225^Ac-L1 in PSMA+ RENCA without or with blockade and RENCA (wt) cells at 37°C.

We characterized RENCA (wt) and PSMA+ RENCA cell lines by studying flow cytometry, immunoblotting, and RT-PCR assays for PSMA cell-surface, protein, and gene expression, respectively, as shown in **Figures 1A–D**. PSMA levels in the PSMA+ RENCA cells were significantly lower than PSMA+ PC3 PIP and higher than the LNCaP and PSMA− PC3 flu cell lines used in the same assay (**Supplementary Figure 1**). The receptor numbers per cell for RENCA (wt) and PSMA+ RENCA cells were 1.5 × 10^3^ and 1.1 × 10^6^, respectively, compared to PSMA+ PIP (3.9 × 10^6^), LNCaP-FGS (5.6 × 10^3^), and PSMA− PC3 flu cells (0.1 × 10^3^), respectively. The higher PSMA protein expression of the PSMA+ RENCA cells compared to the RENCA (wt) cells was validated in WB (**Figures 1B, C**) and RT-PCR (**Figure 1D**), respectively. PSMA gene expression in PSMA+ RENCA cells was ~ 20-fold higher than in RENCA cells.

### 3.2 ^68^Ga-L1, ^177^Lu-L1, and ^225^Ac-L1 display high and specific uptake in PSMA+ RENCA cells

The structures of the radiotracers, ^68^Ga-L1, ^177^Lu-L1, and ^225^Ac-L1, are shown in **Figure 1E**. The molar activities of ^68^Ga-L1 were > 74 MBq/nmol, ^177^Lu-L1 ≥ 37 MBq/nmol, and ^225^Ac-L1 > 8.9 MBq/nmol, respectively. Cell uptake studies using ^68^Ga-L1/^177^Lu-L1/^225^Ac-L1 showed higher uptake in PSMA+ RENCA cells than the RENCA (wt) cells, as shown in **Figures 1F–H** and **Supplementary Table 1**. Blocking studies revealed a significant (*p* < 0.001) lowering of uptake upon coincubation with ZJ43 (PSMA blockade, 10 µM), indicating the binding specificity of the agents. For all radiotracers, uptake was significantly higher in PSMA+ RENCA cells than in the RENCA (wt) cells, as anticipated. Additionally, the uptake of ^68^Ga-L1 (44.6% ± 3.2% at 0.5 h, 49.0% ± 3.6% at 1 h, and 30.9% ± 11.1% at 2 h) in PSMA+ RENCA cells was significantly higher than that of ^177^Lu-L1 (22.1% ± 0.5% at 1 h, 23.8% ± 0.5% at 4 h, and 26.3% ± 3.1%) and ^225^Ac-L1 (4.1% ± 0.2% at 1 h, 11.2% ± 3.2% at 4 h, and 17.1% ± 1.5% at 24 h), respectively.

### 3.3 *In vivo* characterization

#### 3.3.1 ^68^Ga-L1/^225^Ac-L1 demonstrates PSMA-specific uptake in PSMA+ RENCA models

**Figure 2:**
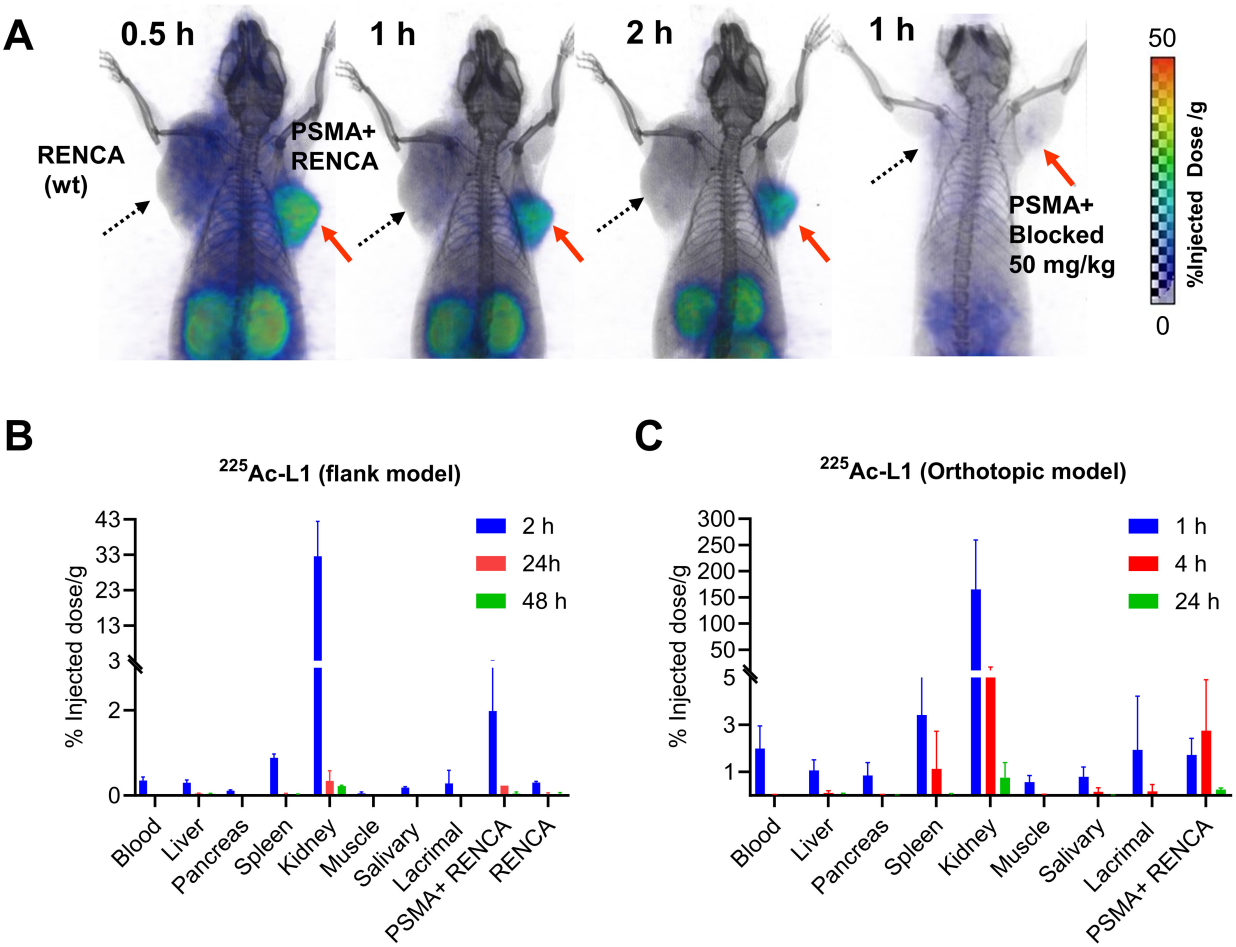
**(A)** PSMA PET-CT imaging in murine renal cell carcinoma, PSMA+ RENCA (right flank), and RENCA (left flank) with ^68^Ga-L1. Male BALB/c tumor-bearing mice were injected with 7.4 MBq of ^68^Ga-L1 through the tail vein, and PET-CT images were acquired 0.5 h, 1 h, and 2 h postinjection. Red arrow: PSMA+ RENCA; dotted black arrow: RENCA (wt). **(B)** Tissue biodistribution data for ^225^Ac-L1 in flank models bearing PSMA+ RENCA (right) and RENCA (wt) (left). **(C)** Tissue biodistribution data for ^225^Ac-L1 in orthotopic models bearing PSMA+ RENCA in the right kidney. Data, mean ± SEM of three mice.

PET/CT imaging demonstrated significantly higher tumor uptake of ^68^Ga-L1 in the PSMA+ RENCA tumors than the RENCA (wt) tumors, as shown in **Figure 2A**. The agent displayed the highest tumor uptake at 0.5 h and fast clearance after 1 h after injection. The kidneys were the only visible organ showing a high accumulation of ^68^Ga-L1. A significantly reduced tumor and kidney uptake were noted after coinjection with ZJ43, suggesting PSMA-specific binding of the agent.

The tissue biodistribution study of ^225^Ac-L1 (**Figure 2B**, **Supplementary Table 2**) revealed that PSMA+ RENCA tumor uptake was low (2.01%ID/g ± 1.07%ID/g at 2 h) and washed out by 24 h (0.23%ID/g ± 0.03%ID/g) and 48 h (0.04%ID/g ± 0.04%ID/g). Furthermore, kidney uptake was high at 32.53%ID/g ± 9.93%ID/g at 2 h, with fast clearance observed at 0.34%ID/g ± 0.24%ID/g at 24 h and 0.22%ID/g ± 0.02%ID/g at 48 h. PSMA blocking using ZJ43 (50 mg/kg) significantly decreased uptake in PSMA+ RENCA tumors (0.45%ID/g ± 0.11%ID/g) and kidneys (1.23%ID/g ± 0.34%ID/g). Kidney uptake is anticipated to be primarily due to the clearance of the agent, specifically at the early time points, and partially due to the high PSMA expression in murine renal cortical proximal tubules (37). An additional biodistribution study was done using orthotopic tumors at 1 h, 4 h, and 24 h postinjection. The observed tumor uptake was in the same range as the flank model. A high kidney uptake was noted at 1 h, 165.25%ID/g ± 94.23%ID/g, and fast clearance, 10.22%ID/g ± 6.77%ID/g at 4 h and after 24 h (0.76%ID/g ± 0.63%ID/g) postinjection, respectively (**Figure 2C**, **Supplementary Table 3**).

#### 3.3.2 ^68^Ga-L1 PET/MR imaging detects spontaneous lung metastases from PSMA+ RENCA models in orthotopic implantation

**Figure 3:**
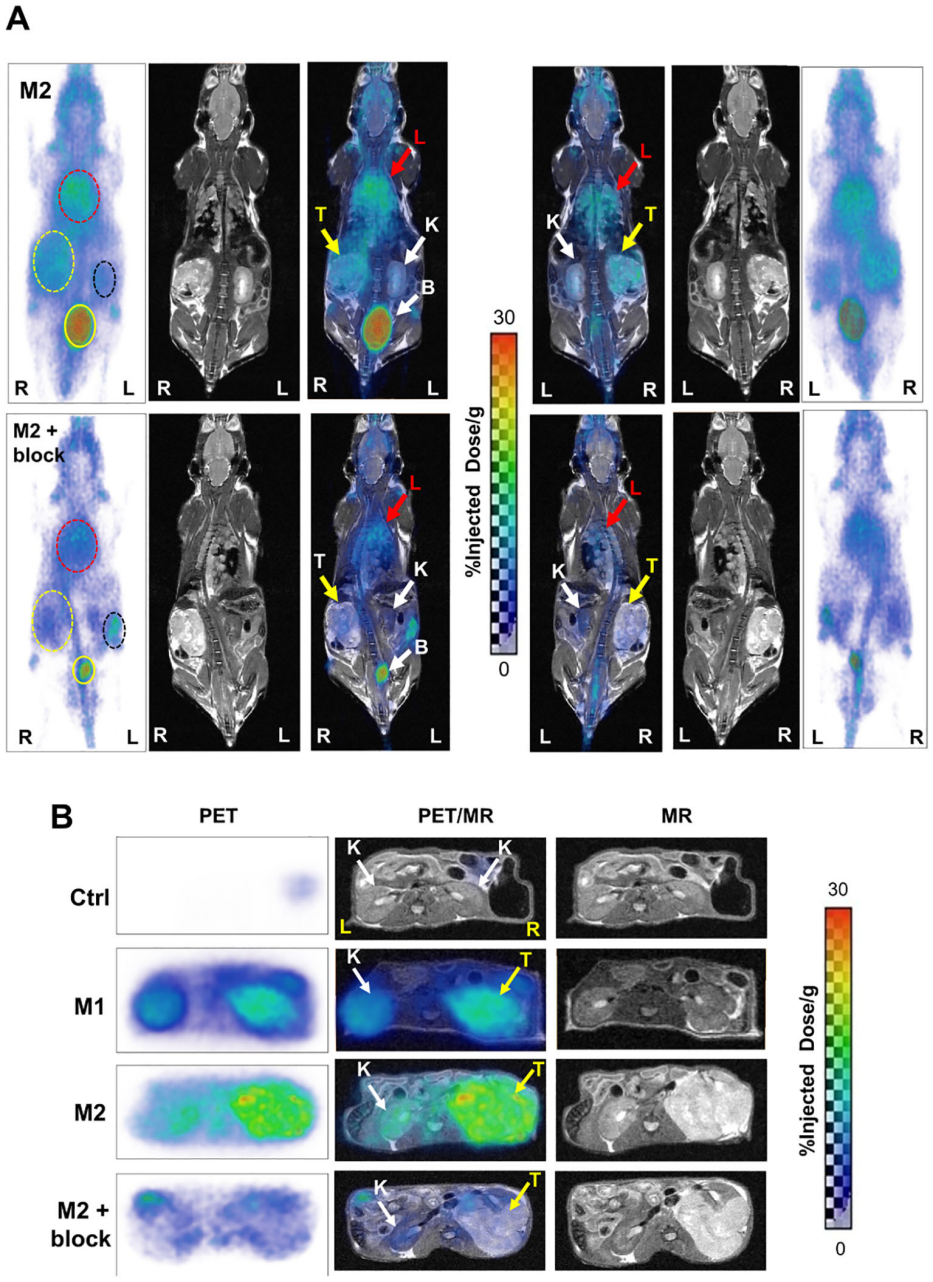
**(A)** Whole-body PET/MR images of PSMA+ RENCA orthotopic tumor-bearing mice: M2 (32 days post-tumor inoculation; top panel) and M2 + blocker (34 days post-tumor inoculation; coinjection with PSMA-targeted ZJ43; middle panel) in prone (left side) and supine view (right side) at 3 h postinjection of 68Ga-L1. K, kidney (black dotted circle, white arrow); T, orthotopic tumor (yellow dotted circle, yellow arrow); L, lung (red dotted circle, red arrow); B, bladder (yellow solid circle, white dotted arrow); R, right; L, left. All images are decay-corrected and adjusted to the maximum value. **(B)** Axial PET (left panel), fused PET/MR (middle panel), and T2-weighed MR (right panel) images of Ctrl (tumor-free; *top panel*) and RENCA-orthotopic tumor-bearing mice (M1, 21 days posttumor inoculation; M2, 32 days posttumor inoculation; and M2 with blocker, 34 days posttumor inoculation) at 3 h postinjection of ^68^Ga-L1.

^68^Ga-L1 was also evaluated in PSMA+ RENCA orthotopic tumors at 21 days after tumor inoculation, as shown in **Figure 3A** (coronal view) and **Supplementary Figure 2**. Clear tumor delineation was noted in the axial view at 3 h postinjection (**Figure 3B**). Kidney uptake was high initially (**Supplementary Figure 2**) and displayed fast clearance, demonstrating the highest tumor-to-kidney uptake ratio at 3 h postinjection (**Figure 3B**). A significantly high lung uptake was noted during 1–5 h postinjection due to spontaneous lung metastases at orthotopic implantation (*n* = 3). Whole-body coronal PET imaging in **Figure 3A** demonstrated a specific and high accumulation of ^68^Ga-L1 in the tumor-bearing right kidney and the right lobes of the lungs. One mouse (M2) underwent a second imaging study session on day 32 to evaluate disease progression. An intra-animal blocking study of the same mouse was conducted on day 34 to confirm PSMA-specific uptake in the tumors and lung metastases using ZJ43 (50 mg/kg). Significant blocking was observed in the primary tumor and partial blocking in the lungs (**Supplementary Figure 3**).

**Figure 4:**
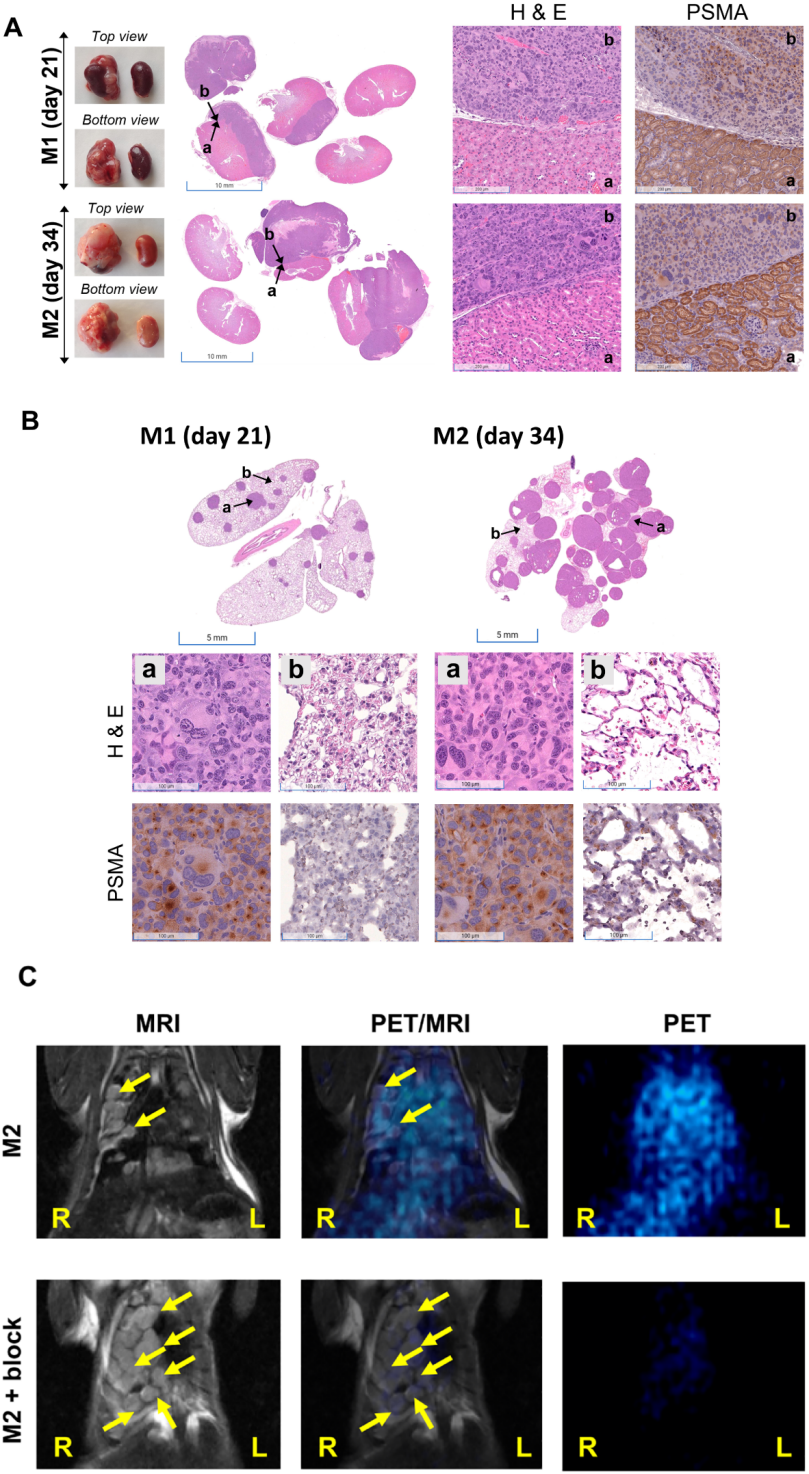
**(A)** Representative photographs of kidneys (*left panel*) and H&E staining of kidney sections (*middle panel*, scale bar, 5 mm) in orthotopic RENCA tumor-bearing mice (M1 and M2) harvested at the times indicated. Histopathological analysis of tumor-bearing kidneys by H&E and PSMA staining (scale bar, 100 µM; ×20). Areas characterized by intense purple staining (H&E) indicate the presence of renal tumors and demonstrate lower PSMA staining. **(B)** Representative images (scale bar, 100 µM; ×20) of H&E (*middle panel*) and PSMA (*bottom panel*) staining observed in nonmetastatic and micrometastatic regions of the lungs (represented by dense purple staining in H&E) were indicated by b and a, respectively, in the top panel. High PSMA expression was observed in the micrometastatic sites. **(C)** Coronal view PET/MRI imaging of the lungs of M2 showing high soft tissue contrast in MR imaging and high sensitivity of PET imaging.

Furthermore, disease progression was demonstrated in **Figure 3B** (axial view). Compared to the control mouse from the same cohort, M1 (imaged on day 21 after tumor inoculation and euthanized for histological characterization) and M2 (on day 32) showed higher ^68^Ga-L1 in the right kidney area (**Figure 3B**; **Supplementary Figure 3**). The tumor-free control mouse also had no lung metastases, as shown in **Supplementary Figure 4**. Histopathological and IHC characterization was conducted to confirm PSMA positivity in the kidneys, lungs, and PSMA+ RENCA tumors (**Figures 4A, B**). MR imaging at day 21 posttumor inoculation detected lower volume lung metastases than day 32; H&E data further confirmed lung metastases in those areas (**Figure 4B**). Additionally, PSMA expression was significantly higher in the lung metastases than in the primary tumors on days 21 and 34, confirming the higher accumulation of ^68^Ga-L1 in the metastatic diseases compared to the primary tumors (**Figures 4B, C**). Furthermore, the high-resolution MR imaging (**Figure 4C**) enabled the detection of metastatic lesions in the lung area. Many of those lesions in MR imaging were correlated with ^68^Ga-L1 uptake in PET imaging. We did not observe any liver metastases from this mouse cohort until euthanization on day 34 for IHC characterization (**Supplementary Figure 5**).

### 3.4 Radiopharmaceutical therapy

#### 3.4.1 ^225^Ac-L1 displayed dose-dependent cytotoxicity in PSMA+ RENCA cells and tumor models

**Figure 5:**
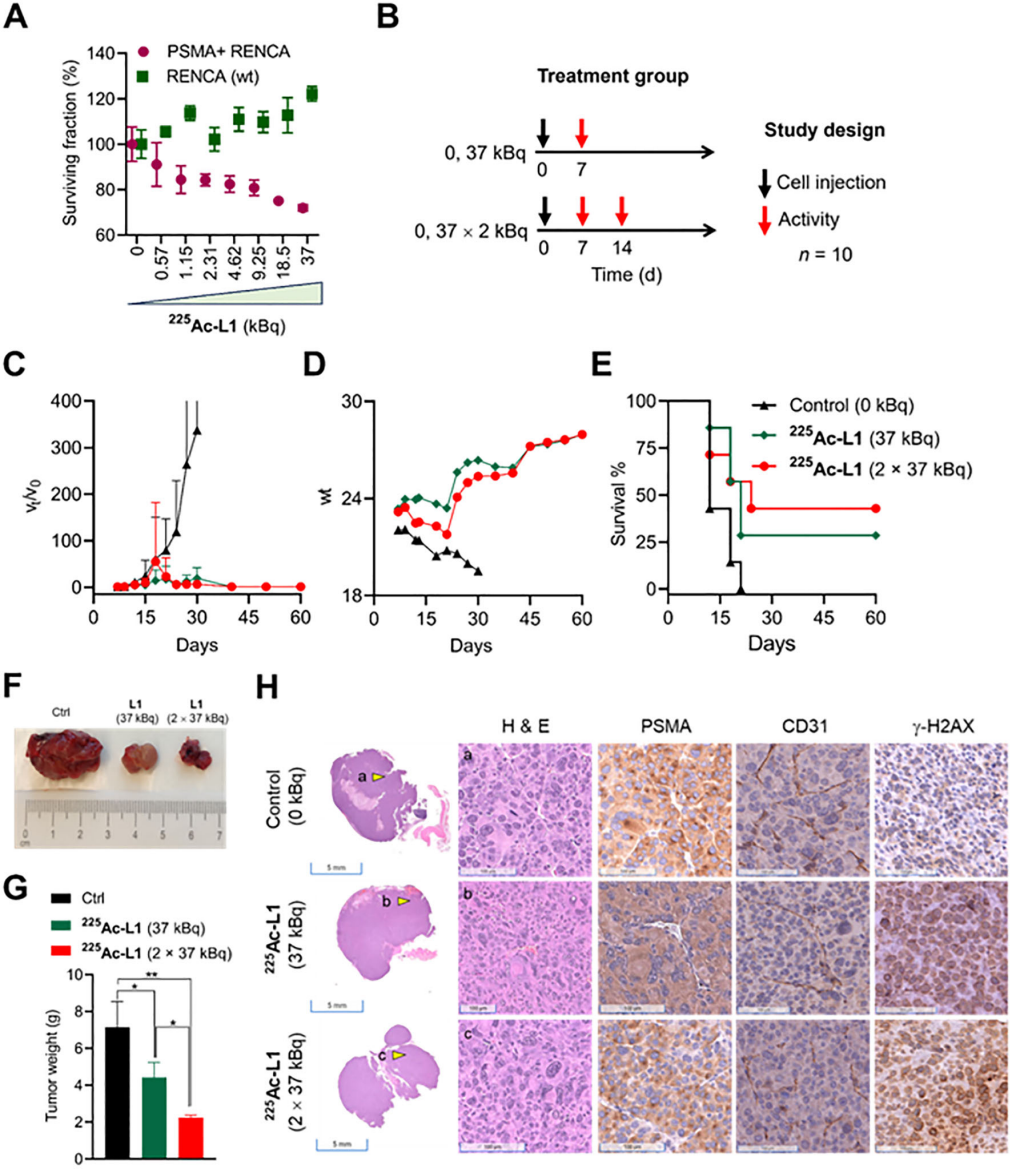
**(A)** Cell viability was assessed with ^225^Ac-L1 in a dose-dependent manner (0–37 kBq) against PSMA+ (R+) and RENCA (wt) (R-) cells by a cell Titer-Glo luminescent cell viability assay. **(B)** Study design to evaluate the treatment effect in PSMA+ RENCA flank tumors following administration of ^225^Ac-L1 via tail-vein injection. **(C)** Relative tumor volume (*v_t_*/*v_o_*); tumor growth curves relative to tumor volume at day 7 (set to 1). **(D)** Body weights of control and treatment groups. **(E)** The Kaplan–Meier curve revealed significant tumor growth control in the RENCA xenograft model for the treatment groups (*p* < 0.03) compared to the untreated control group. **(F)** Representative photographs were collected on day 18 after the tumor inoculation from Ctrl (0 kBq) and treatment groups (^225^Ac-L1; 37 kBq and 2 × 37 kBq). **(G)** The average weight of tumors collected from Ctrl (0 kBq) and treatment groups (^225^Ac-L1; 37 kBq and 2 × 37 kBq). **(H)** Representative H&E-stained PSMA+ RENCA tumor sections (*left first panel*; scale bar, 5 mm; ×0.2) from control and treatment groups. Representative images (scale bar, 100 µM; ×20) of H&E (left second panel), IHC of PSMA (middle panel), CD31 (right third panel), and γ-H2AX (right last panel) staining, respectively, observed in PSMA+ RENCA tumors indicated by a yellow shaded triangle in the left panel from the control and treatment groups.

A dose-dependent cell viability assay was conducted to evaluate the cytotoxicity of ^177^Lu-L1 and ^225^Ac-L1 in the studied cell lines, as shown in **Supplementary Figure 6** and **Figure 5A**, respectively. ^177^Lu-L1 did not display any decrease in cell viability up to 144 h postincubation (0–3,700 kBq). PSMA-specific dose-dependent lowering in cell viability was observed with ^225^Ac-L1 (0–37 kBq) in PSMA+ RENCA cells after 48 h incubation, suggesting higher efficacy of short-range, high-energy α-particle-emitting ^225^Ac-L1 compared to long-range, low-energy β-particle-emitting ^177^Lu-L1. Accordingly, a treatment study was conducted using ^225^Ac-L1 in the RENCA flank model (average tumor volume of ≤ 20 mm^3^) after 1 week of tumor inoculation (**Figures 5B–D, F**). The doses were selected based on our reported long-term toxicity study (28). Tumor-bearing mice (*n* = 10) were randomized for tumor size, and receiving any doses of 37 kBq demonstrated significant growth delay in tumors (*p <* 0.005) compared to the control group treated with saline. Three mice from each group were removed from the study for histopathological evaluation after 8 days after the dose administration. All control mice were euthanized within 18 days posttreatment because of exponential tumor growth (> 1,000 mm^3^) (**Figure 5C**). The surviving mice from the treatment groups (two mice from the 37-kBq group and three mice from the 2 X 37 kBq) remained tumor-free up to 120 days. The treated mice displayed increased body weight 2–3 weeks posttreatment (**Figure 5D**). The average median survival for the control group was 12 days, significantly lower (*p* < 0.03) than the treatment groups, 21 days (37 kBq) and 24 days (2 X 37 kBq), respectively (Log-rank [Mantel–Cox] test). The average median survival was not statistically different (*p* = 0.67) among the treatment groups (**Figure 5E**). Furthermore, treatment with 225Ac-L1 (2×37 kBq) led to a significant reduction in tumor growth compared to the control group (p < 0.01) and the 225Ac-L1 (37 kBq) group (p < 0.05) (**Figures 5F**, **G**). IHC characterization of CD31 (tumor neovasculature), PSMA, and γ-H2AX (DNA double-strand break) was conducted from the cohorts after 8 days posttreatment (**Figure 5H**). While a moderate decrease was noted in PSMA and CD31 expression, significantly higher staining of γ-H2AX was observed in the treated tumor tissues compared to control untreated tumors, suggesting DNA double-strand damage by α-particle-emitting ^225^Ac-L1.

## 4 Discussion

The emergence of effective and generally well-tolerated immunotherapies has propelled advancements in therapy for patients with mRCC. While immunotherapy may offer clinical benefits, only a subset of patients respond durably to those treatments. Furthermore, there are only limited options for treatment-resistant mRCC. Radiotheranostic agents have garnered increased attention recently. While being targeted, such agents also benefit from having few and generally mild off-target effects in practice (38, 39). Consequently, several radiotheranostic agents have received FDA approval for various indications, including Pluvicto™ (^177^Lu-PSMA-617), Xofigo^®^ (^223^RaCl_2_), and LUTATHERA (^177^Lu-DOTATATE) (40, 41). Radiotheranostic agents are relatively less studied in metastatic RCC. One notable agent under investigation is the chimeric antibody girentuximab (cG250), which targets carbonic anhydrase IX (CAIX), a protein overexpressed in more than 90% of ccRCC cases. Immuno-PET/single-photon emission computerized tomography (SPECT) of ^124^I- and ^111^In-labeled analogs of cG250 and radioimmunotherapy of ^131^I- and ^177^Lu-labeled analogs are in phase III clinical trials (42–48). However, overall efficacy and diagnostic outcomes have not been impressive because of the slow pharmacokinetic profile and poor tumor penetration of cG250 (MW 150 kDa) in solid tumors, as well as severe myelotoxicity (45, 46). We and others have developed CAIX-based low molecular weight agents; however, clinical studies are currently focused on studying the imaging aspects of agents (49–52).

There are several key findings from this work: First, to facilitate our studies, we conducted a detailed characterization of PSMA+ RENCA and RENCA (wt) cells and tumors while comparing with our standard prostate cancer cell lines, PSMA+ PC3 PIP and LNCaP, and PSMA− PC3 flu cells. The data revealed that the RENCA (wt) cells are associated with lower PSMA expression than the PSMA+ RENCA cells but significantly higher than the PC3 flu cells. Second, cell uptake studies using ^68^Ga-L1/^177^Lu-L1/^225^Ac-L1 confirmed that the PSMA expression levels are sufficient for PSMA-specific binding of the cells. Low cell and tumor uptake of ^225^Ac-L1 could be associated with the low specific activity we used for the studies. Additionally, fast-growing PSMA+ RENCA tumors were associated with large necrotic areas and low PSMA expression, as shown in **Figure 4A** within 3–4 weeks after tumor inoculation. Third, ^68^Ga-L1 PET/CT and ^68^Ga-L1 PET/MR imaging in flank and orthotopic tumors were studied to evaluate tumor uptake and retention. In our experience, PET/CT was unsuitable for locating orthotopic tumors in CT. Furthermore, contrast-enhanced CT using diatrizoate sodium (meglumine) in the orthotopic model resulted in negative tumor contrast (data not shown) and was associated with additional toxicities. High resolution and superior soft tissue contrast from MR imaging enabled reliable tumor and metastasis detection in the kidneys and lungs, respectively, as shown in **Figures 3**, **4C**.

Fourth, the PSMA+ RENCA, a human PSMA-transduced tumor model, is considered a limitation of our approach to mimicking neovascular PSMA expression in RCC. Despite its limitations, the PSMA+ RENCA model holds clinical relevance due to its similarity to RCC metastasis patterns. The lung is the most common metastatic site for patients with RCC, as observed in the orthotopic PSMA+ RENCA model (53). The low uptake of ^68^Ga-L1 and ^225^Ac-L1 and low PSMA expression (by IHC) associated with the PSMA+ RENCA model could be related to the highly vascular nature of this model. The observed low tumor uptake and fast clearance of ^225^Ac-L1 from PSMA+ RENCA will likely mirror neovascular PSMA expression in the context of radiopharmaceutical therapy. Further biological studies are currently ongoing in our lab to address this issue. Notably, the RENCA model is the most used preclinical tumor for studying the efficacy of TKIs and immunotherapy with ICIs and in various combination treatment regimens; however, its morphology does not resemble clear cell RCC subtypes (54–56). The developed immunocompetent PSMA+ RENCA model will be useful for studying PSMA-based radiopharmaceutical therapy in similar contexts.

Fifth, the difference in radiotoxicity of ^177^Lu-L1 compared to ^225^Ac-L1 in the cell viability assay can be explained by the distinct radiation properties emitted by each radionuclide. Specifically, ^177^Lu emits β-particles with long-range (~ 1.8 mm) and low linear energy transfer (LET) (~ 0.2 keV/µm) radiations, whereas α-emitting ^225^Ac is associated with short-range (50–100 µm) and high LET radiations (~ 80 keV/µm). We previously demonstrated higher efficacy of ^225^Ac-L1 (28) and ^212^Pb-based α-particle-based radiopharmaceutical therapy (29) compared to ^177^Lu-L1 in cells and treating micrometastatic diseases. This suggests that α-particle-emitter radiopharmaceutical therapy could be a superior option for mRCC. ^225^Ac-labeled vascular-targeted radioimmunotherapy exhibited significant efficacy in solid tumors in preclinical and clinical studies (57, 58). Compared to antibodies, low-molecular-weight agents, as we studied here, have the potential for delivering higher tumor-targeting radiation due to their rapid tumor-targeting, short blood half-life, and high tumor diffusibility.

The development of ICIs has remarkably changed the treatment paradigm of RCC, although many patients do not respond reliably using a standalone treatment approach. Consequently, efforts are underway to enhance the efficacy of ICIs by creating a more immunogenic tumor microenvironment. Radiotherapy, among other modalities, has emerged as a promising candidate for combination therapy with ICIs, despite its limited role in metastatic settings (59, 60). Targeted radiopharmaceutical therapy employing β- and α-particle emitters holds potential to address these challenges. Moreover, radiopharmaceutical therapy is known to augment the immunogenicity of tumors by inducing inflammation within the tumor microenvironment (61–66). While the immunomodulatory effects of β- and α-emitting radiotherapeutics were studied in prostate cancers in preclinical and clinical settings, these effects are yet to be tested in the tumor microenvironment of mRCC (62, 63, 65, 66). The data from our proof-of-concept study is promising in this context, warranting further investigation to evaluate the mechanism of action using ^177^Lu-L1 and ^225^Ac-L1 in standalone and in combination with standard-of-care ICIs. The well-established understanding of ICIs and Pluvicto™ (^177^Lu-PSMA-617) in patient studies provides a solid foundation for further exploration of investigational agents such as ^177^Lu-L1 and ^225^Ac-L1 studied here in mRCC. Extrapolating from the promising preclinical results described herein, we suggest PSMA-targeted radiotheranostics as another option to treat patients with mRCC as a standalone therapy or in combination with ICIs or other immunomodulatory approaches.

## Data Availability

The original contributions presented in the study are included in the article/**Supplementary Material**. Further inquiries can be directed to the corresponding author.
