# Supplementary material for: Performance of PSMA-targeted radiotheranostics in an experimental model of renal cell carcinoma
Source: Front Oncol. 2024 Sep 10;14:1432286. doi: 10.3389/fonc.2024.1432286 (PMC11423292; doi:10.3389/fonc.2024.1432286)
Supplement: Supplementary file 1 [file DataSheet1.pdf]

# *Supplementary Material*

**Rajan Singh[1], Anand K. Thotakura[1], Suresh Alati[1], Alla Lisok[1], Zirui Jiang[1], Vanessa F. Merino[1], Santosh Yadav[1], Yasser Ged[2,3], Nirmish Singla[2,3], Lilja Slones[1,2], Michael Gorin,[5] Martin G. Pomper[4], Steven P. Rowe[6], Sangeeta Ray Banerjee[1,2]**

[1]Russell H. Morgan Department of Radiology and Radiological Science, [2]Sidney Kimmel Comprehensive Cancer Center, [3]Department of Urology and Oncology, Brady Urological Institute, Johns Hopkins University, Baltimore, MD, USA, [4]Department of Radiology, UT Southwestern Medical Center, Dallas, TX, USA, [5]Department of Urology, Icahn School of Medicine at Mount Sinai, New York, NY, USA, [6]Department of Radiology, University of North Carolina, Chapel Hill, NC, USA.

*** Correspondence:** Sangeeta Ray Banerjee; email:sray9@jhmi.edu

# Supplementary Figures

**Supplementary Figure 1.** Flow cytometry of PSMA surface expression in RENCA (wt), PSMA+ RENCA, PSMA- flu, PSMA+ PC3 PIP and LNCaP cell lines.

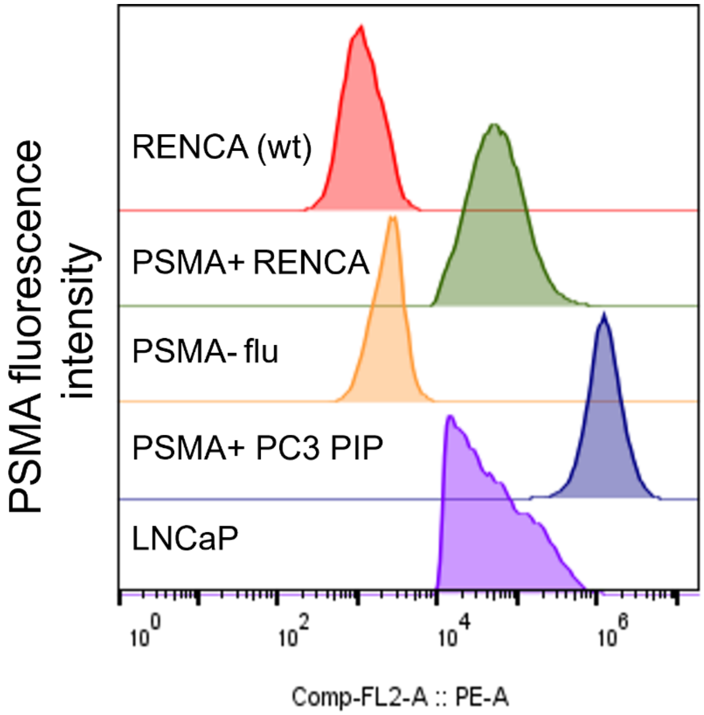

**Supplementary Figure 2.** Whole-body PET/MR images of RENCA-orthotopic tumor-bearing mice (M1) at 1 h (*top panel*), 3 h (*middle panel*) and 5.5 h (*bottom panel*) post-injection of $^{68}$Ga-L1. Uptake in the kidneys, tumors, and lungs is indicated by solid white, yellow, and red arrows, respectively. K = kidney (black dotted circle, white arrow); T = orthotopic tumor (yellow dotted circle, yellow arrow); L = lung (red dotted circle, red arrow); B = bladder (yellow solid circle, white arrow); U = ureter, R = right; L = left. All images are decay-corrected and adjusted to the same maximum value.

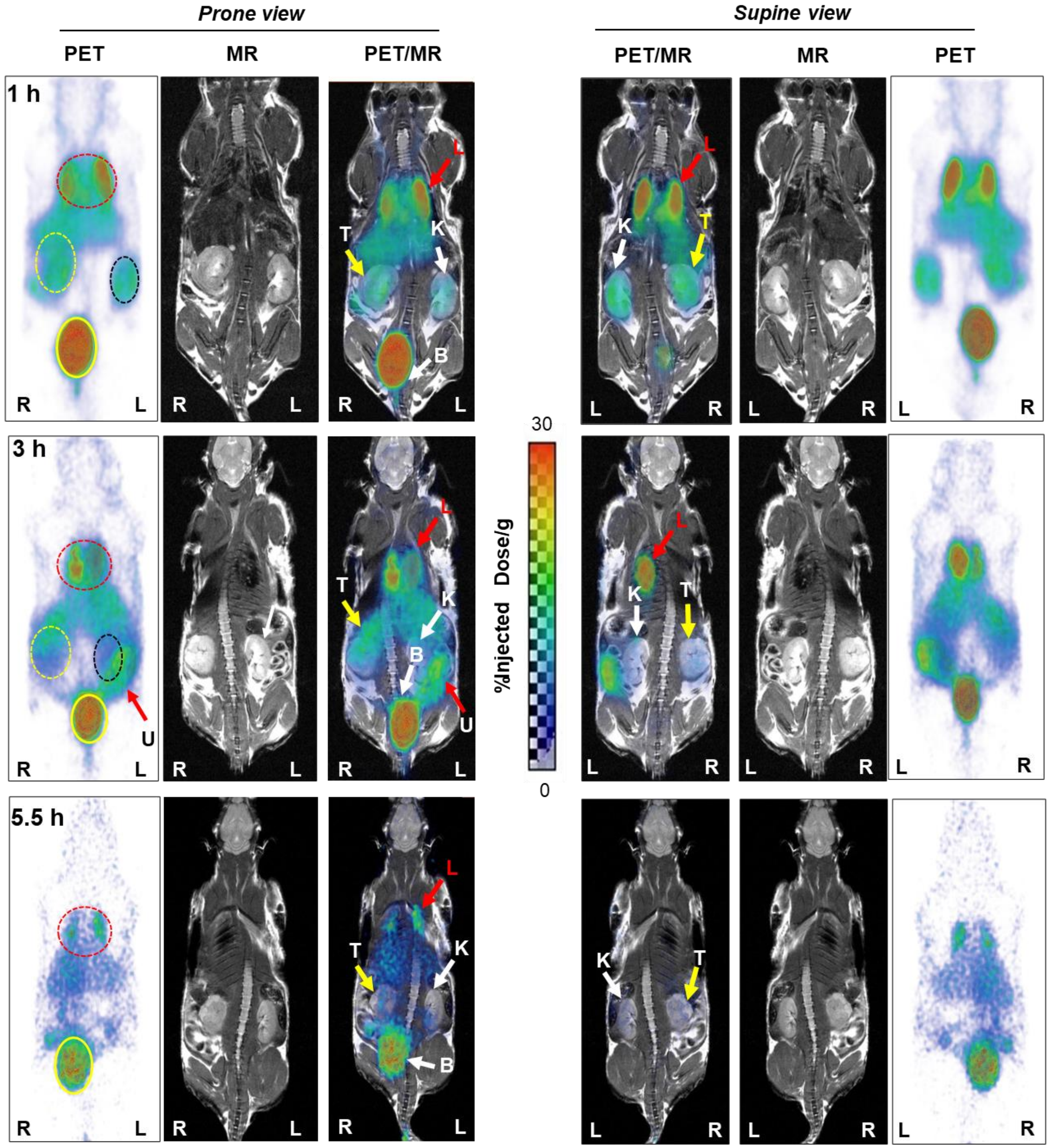

**Supplementary Figure 3.** (A-C) Uptake of $^{68}$Ga-L1 at 3 h post-injection in (A) right kidney/tumor-bearing kidney, (B) left healthy kidney, and (C) lungs measured by ROI analysis using AMIDE software. Statistical significance was determined using a two-tailed unpaired *t*-test with Welch's correction (ns indicate not significant, * $P \leq 0.05$, ** $P \leq 0.01$, and *** $P \leq 0.001$).

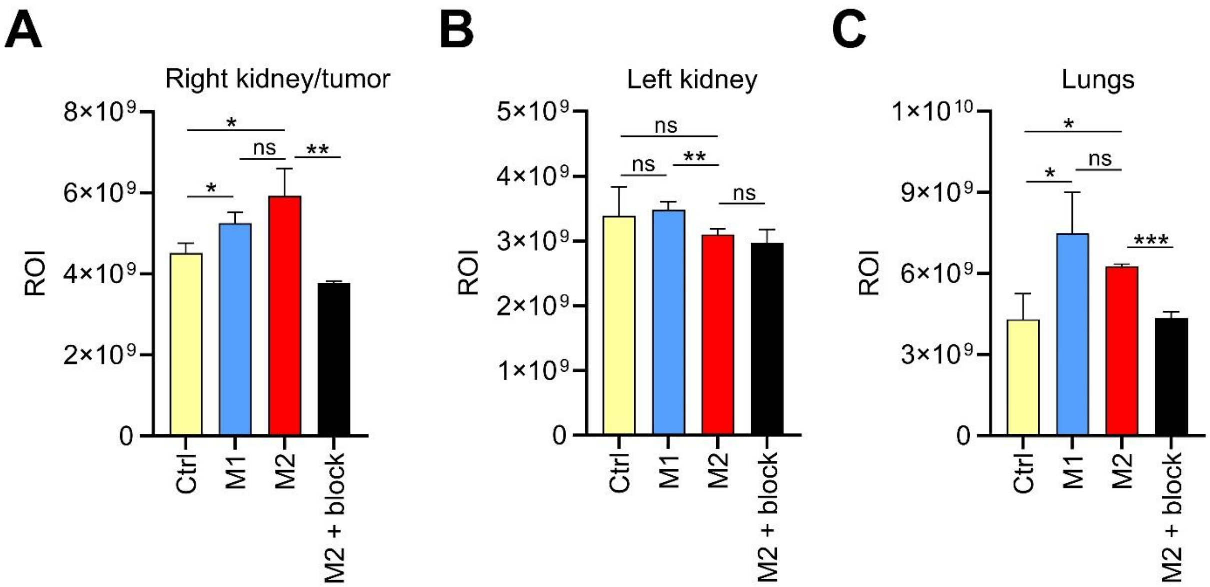


**Supplementary Figure 4**. (A) Representative photograph of H&E-stained lung sections (*left panel*, scale bar, 5 mm) in tumor-free control mouse harvested at day 32 from the same cohort of mice. Histopathological analysis of lungs by H&E (scale bar, 100 μM; ×20) indicated by a yellow-filled triangle.

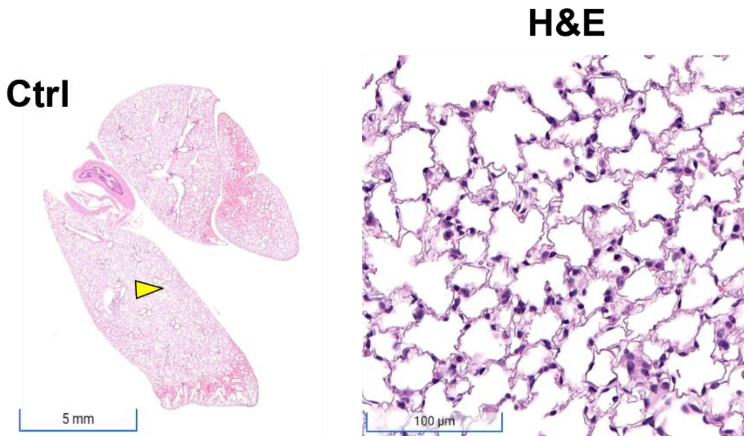

**Supplementary Figure 5. (**A) Representative photographs of H&E-stained liver sections (*left panel*, scale bar, 10 mm) in orthotopic RENCA tumor-bearing mice (M1 and M2) harvested at the times indicated. Histopathological analysis of liver by H&E and PSMA-staining sections (scale bar, 100 μM; ×20) indicated by yellow filled triangles.

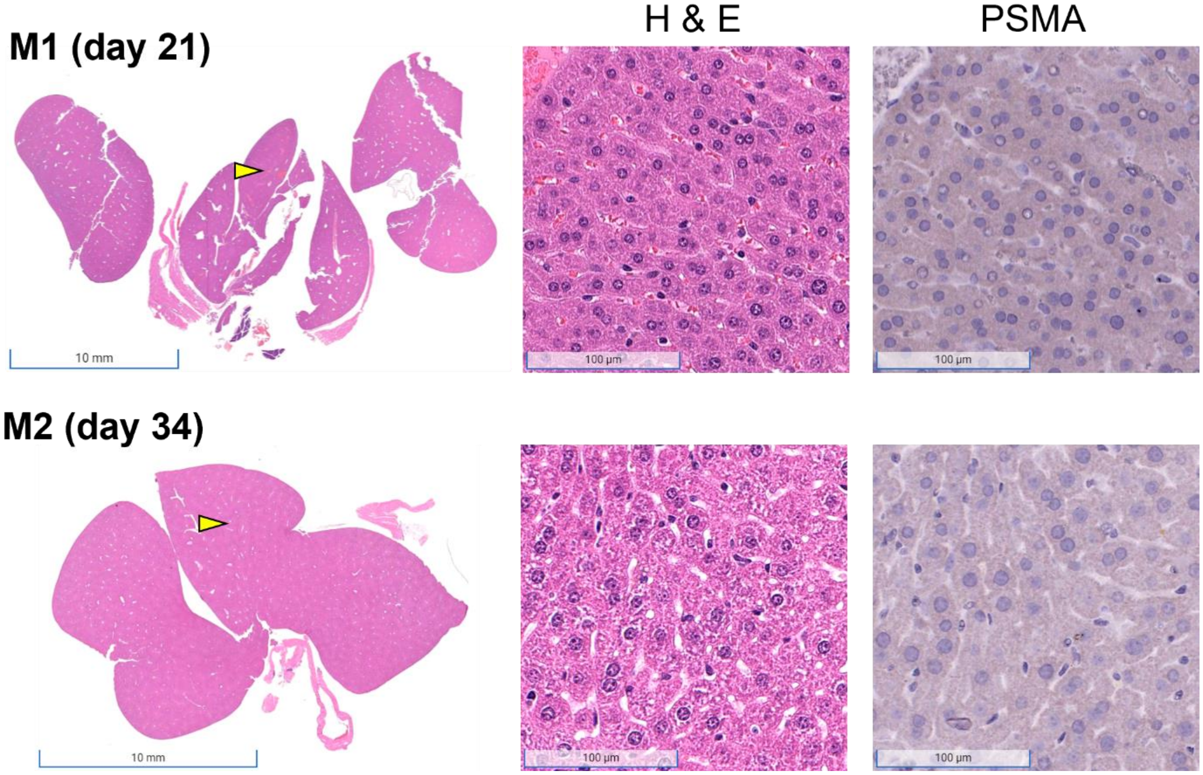


**Supplementary Figure 6.** Cell viability was assessed for 96 h incubation at 37 °C with $^{177}$Lu-L1 in a dose-dependent manner (0-3700 kBq) against PSMA+ RENCA and RENCA (wt) cells by cell Titer-Glo luminescent cell viability assay**.**

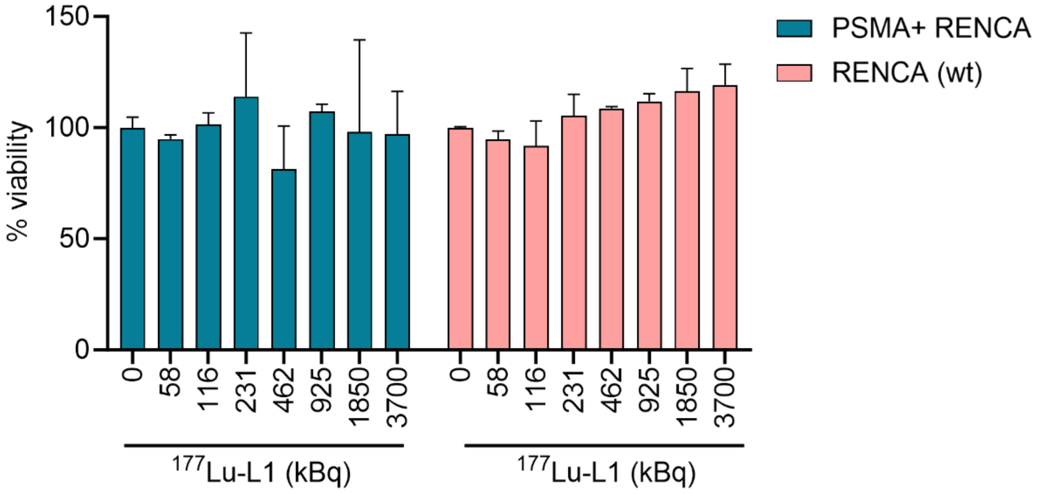

# Supplementary Tables

**Supplementary Table 1.** Cell uptake of the selected compounds in PSMA+ RENCA and RENCA (wt) cells. ZJ43, a known PSMA inhibitor, was used in a final concentration of 10 µM for blocking studies. (data expressed in % incubated dose per 1 × $10^6$ cells).

| Radioisotope | Cells | **Incubation time** | | |
|---|---|---|---|---|
| | | **0.5 h** | **1 h** | **2 h** |
| **$^{68}$Ga-L1** | PSMA+ RENCA | 44.6 ± 3.2 | 49.0 ± 3.6 | 30.9 ± 11.1 |
| | PSMA+ RENCA blockade | 23.6 ± 2.3 | 25.5 ± 0.5 | 12.8 ± 3.0 |
| | | **1 h** | **4 h** | **24 h** |
| **$^{177}$Lu-L1** | PSMA+ RENCA | 22.1 ± 0.5 | 23.8 ± 0.5 | 26.3 ± 3.1 |
| | PSMA+ RENCA blockade | 1.0 ± 0.4 | 0.9 ± 0.2 | 1.3 ± 0.0 |
| | RENCA (wt) | 1.2 ± 0.4 | 1.2 ± 0.5 | 0.8 ± 0.5 |
| | | **1 h** | **4 h** | **24 h** |
| **$^{225}$Ac-L1** | RENCA PSMA+ | 4.1 ± 0.2 | 11.2 ± 3.2 | 17.1 ± 1.5 |
| | PSMA+ RENCA blockade | 2.4 ± 0.3 | 2.7 ± 0.7 | 4.3 ± 0.8 |
| | RENCA (wt) | 2.2 ± 0.2 | 2.4 ± 0.3 | 8.2 ± 1.4 |

**Supplementary Table 2**. Tissue biodistribution data for $^{225}$Ac-L1 in flank models bearing PSMA+ RENCA (right) and RENCA (left)

| Tissue | **2 h** | **24 h** | **48 h** |
|---|---|---|---|
| Blood | 0.35 ± 0.09 | - | - |
| Liver | 0.30 ± 0.06 | 0.04 ± 0.01 | 0.04 ± 0.01 |
| Pancreas | 0.11 ± 0.02 | - | - |
| Spleen | 0.89 ± 0.09 | 0.03 ± 0.02 | 0.02 ± 0.01 |
| Kidney | 32.53 ± 9.93 | 0.34 ± 0.24 | 0.22 ± 0.02 |
| Muscle | 0.05 ± 0.03 | - | - |
| Salivary | 0.18 ± 0.02 | 0.01 ± 0.00 | - |
| Lacrimal | 0.29 ± 0.31 | - | - |
| PSMA+ RENCA | 2.01 ± 1.07 | 0.23 ± 0.00 | 0.04 ± 0.04 |
| RENCA (wt) | 0.30 ± 0.03 | 0.03 ± 0.03 | 0.04 ± 0.04 |

**Supplementary Table 3**. Tissue biodistribution data for $^{225}$Ac-L1 in orthotopic models bearing PSMA+ RENCA in the right kidney. Data, mean ± SEM of 3 mice.

| Tissue | **1 h** | **4 h** | **24 h** |
|---|---|---|---|
| Blood | 2.00 ± 0.95 | 0.02 ± 0.02 | 0.00±0.00 |
| Liver | 1.06 ± 0.45 | 0.12 ± 0.09 | 0.05 ± 0.04 |
| Pancreas | 0.84 ± 0.55 | 0.02 ± 0.02 | 0.01 ± 0.01 |
| Spleen | 3.41 ± 2.24 | 1.13 ± 1.60 | 0.04 ± 0.04 |
| Kidney | 165.25 ± 94.23 | 10.23 ± 6.68 | 0.76 ± 0.63 |
| Muscle | 0.56 ± 0.28 | 0.02 ± 0.03 | - |
| Salivary | 0.78 ± 0.43 | 0.15 ± 0.18 | 0.01 ± 0.01 |
| Lacrimal | 1.94 ± 2.27 | 0.17 ± 0.30 | - |
| PSMA+ RENCA | 1.72 ± 0.71 | 2.74 ± 2.16 | 0.25 ± 0.07 |

**HPLC Methods for $^{68}$Ga-L1,$^{177}$Lu-L1 and $^{255}$Ac-L1**

| Metal | HPLC Retention time | %A (0.1% TFA in Water) | %B (0.1% TFA in ACN) |
|---|---|---|---|
| $^{68}$Ga-L1 | 16.4-18.8 min | 75 | 25 |
| $^{177}$Lu-L1 | 21.2-23.0 min | 79 | 21 |
| $^{225}$Ac-L1 | 26.6-28.4 min | 79 | 21 |

**Radiolabeling Methods**

Eckert and Ziegler $^{68}$Ge/$^{68}$Ga-generator was used as a $^{68}$Ga source. $^{68}$Ga was eluted using 0.5 N HCl solution. TraceSELECT grade water and sodium acetate were used for radiolabeling. Radiolabeling was performed with a minor modification of our reported method (33). To a reaction vial (5 mL) containing 600 µL of $^{68}$Ga(III) (~226 MBq) in 0.1 HCl was neutralized to pH 4 using 15 µL of 5 M ammonium acetate and 10 µL of L1 (2 mM solution). The reaction vial was heated at 95 °C for 10 min. The solution was then cooled, diluted with 200 µl water, and purified by HPLC to provide 172 MBq of $^{68}$Ga-L1. The flow rate was 1 mL/min, with water (0.1% trifluoroacetic acid) (A) and $CH_3CN$ (0.1% trifluoroacetic acid) (B) as the eluting solvents, respectively. An isocratic solution of 75%/25% (A/B) was used to separate the

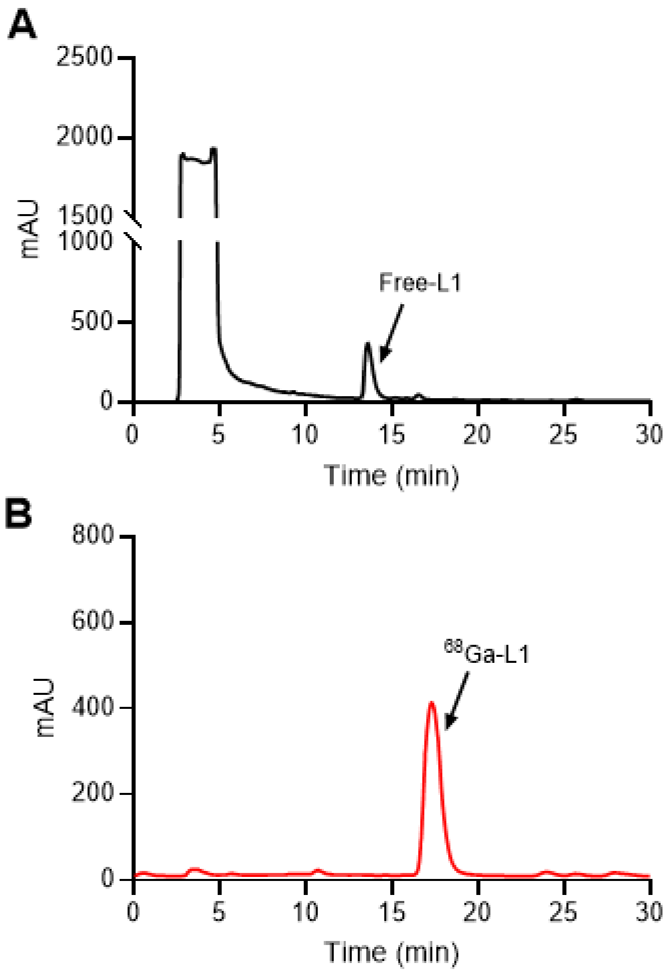


HPLC chromatograms of $^{68}$Ga-L1; A) peak at λ = 220 nm, B) radioactive peak. UV peak at 13.2-14.4 min is related to the unbound ligand L1.

excess ligand from the radiolabeled compound and to ensure the highest purity. HPLC retention time ($t_R$) = 16.4-18.8 min for the radiolabeled product and $t_R$ = 13.2-14.4 min for the free ligand. The acidic eluate was neutralized with 100 μL of 0.1 M $NaHCO_3$ solution, and the volume of the eluate was reduced under vacuum to dryness.

Radiolabeling of $^{177}$Lu-L1 was performed using microwave-assisted synthesis following a previously described protocol (26). Briefly, a solution of $^{177}$Lu (74 MBq in 10 μL of 0.2 M HCl) was added 10 μL of ascorbic acid (1.1 M), about 10 μL of L1 (1 mM), 50 μL of $NH_4OAc$ (0.2 M), to adjust to a pH of about 4. After microwave heating for 6 min, the solution was diluted with 300 μL of water and purified by HPLC. The flow rate was 1 mL/min, with water (0.1% trifluoroacetic acid) (A) and $CH_3CN$ (0.1% trifluoroacetic acid) (B) as the eluting solvents. An isocratic solution of 79% A and 21% B was used to separate excess ligands from the radiolabeled compound and to ensure the highest purity. HPLC retention time ($t_R$) = 21.2-23.0 min for the radiolabeled product and $t_R$ = 33.3-35.5 min for the free ligand. Overall radiochemical yield was ~90% (decay uncorrected), and radiochemical purity was >99.9%.

Radiosynthesis of $^{225}$Ac-L1 was performed using microwave-assisted synthesis following a previously described protocol (27). Briefly, a solution of $^{225}Ac(NO_3)_3$ (3.4 MBq in 10–15 μL of 0.2 M HCl) was added 10 μL of ascorbic acid (1.1 M), about 10 μL of L1 (1 mM), 50 μL of $NH_4OAc$ (0.2 M), and 1–2 μL of $NH_4OAc$ (5 M) to adjust to a pH of about 4. After microwave heating for 5 min at 90°C, the solution was diluted with 0.3 mL of water and purified by HPLC. The flow rate was 1 mL/min, with water (0.1% trifluoroacetic acid) (A) and $CH_3CN$ (0.1% trifluoroacetic acid) (B) as the eluting solvents. An isocratic solution of 79% A and 21% B was used to separate the excess ligand from the radiolabeled compound and to ensure the highest purity. HPLC retention time ($t_R$) = 26.5-28.5 min for

the radiolabeled product and $t_R$ = 33.3-35.5 min for the free ligand. Overall, radiochemical yield was ~90% (decay uncorrected), and radiochemical purity was over 99.9%.
